# Dose and plasma concentration of galantamine in Alzheimer's disease - clinical application

**DOI:** 10.1186/alzrt156

**Published:** 2013-01-03

**Authors:** Carina Wattmo, Erik Jedenius, Kaj Blennow, Åsa K Wallin

**Affiliations:** 1 Clinical Memory Research Unit, Department of Clinical Sciences, Malmö, Lund University, SE-205 02 Malmö, Sweden; 2 Memory Clinic, Skåne University Hospital, SE-205 02 Malmö, Sweden; 3 Alzheimer Disease Research Centre, Department of Neurobiology, Caring Sciences and Society, Karolinska Institute, Hälsovägen 7, SE-141 86 Stockholm, Sweden; 4 Medical Department, Janssen-Cilag AB, Staffansväg 2, SE-192 78 Sollentuna, Sweden; 5 Clinical Neurochemistry Laboratory, Institution of Neuroscience and Physiology, The Sahlgrenska Academy at University of Gothenburg, Sahlgrenska University Hospital, Mölndal, SE-431 80 Mölndal, Sweden

## Abstract

**Introduction:**

Patients with Alzheimer's disease (AD) are currently treated with cholinesterase inhibitors, such as galantamine, without actual knowledge of its concentration in plasma. Our objective was to analyse potential relationships between galantamine concentration, galantamine dose, socio-demographic characteristics, body weight, body mass index (BMI), and treatment response.

**Methods:**

Eighty-four patients with AD recruited from the Memory Clinic, Malmö, Sweden, and treated with galantamine were included in the study. Efficacy measures, including cognition (Mini-Mental State Examination (MMSE), Alzheimer's Disease Assessment Scale - cognitive subscale (ADAS-cog)) and instrumental activities of daily living (IADL), were evaluated at baseline, 2 months after treatment initiation (MMSE only) and semi-annually over 3 years. At these assessments, blood samples were obtained for the analysis of the galantamine concentration, and body weight, BMI, drug dose and time from drug intake were recorded.

**Results:**

All patients had a measurable concentration of galantamine at all assessments. The mean plasma concentration of the drug exhibited a positive linear association with dose (*r *= 0.513, *P *< 0.001). The dose did not differ between sexes. Negative linear associations between the galantamine plasma concentration and BMI (*r *= -0.454, *P *= 0.001) or body weight (*r *= -0.310, *P *= 0.034) were found exclusively in the male group. When mixed-effects models were used, the dose of galantamine (*P *< 0.001), time from drug intake (*P *< 0.001), and BMI (*P *= 0.021) or weight (*P *= 0.002) were factors that predicted the concentration, whereas sex, age, and cognitive and functional changes were not.

**Conclusions:**

High compliance to galantamine treatment was found among all patients in this naturalistic AD study. The impact of BMI or body weight on the plasma concentration of galantamine was important only among males. No relationship was observed between concentration and short-term treatment response or progression rate in terms of cognitive and functional abilities.

## Introduction

Currently, acetylcholinesterase inhibitors (ChEIs) are used as a symptomatic treatment to counteract the progressive and devastating symptoms of Alzheimer's disease (AD). ChEIs prevent the degradation of acetylcholine (ACh) by inhibiting the enzyme acetylcholinesterase (AChE), resulting in increased levels of ACh in the synaptic cleft available for receptor absorption. This enhances cholinergic transmission and improves the communication between neurons [1]. Galantamine is a specific, competitive and reversible ChEI that was approved in Sweden in 2000. Moreover, it is an allosteric modulator at nicotinic cholinergic receptor sites that potentiates cholinergic nicotinic neurotransmission, which provides this ChEI agent with a dual mechanism of action [2]. The half-life of galantamine is 7 to 8 hours. Therefore, to simplify dosing and enhance compliance, a once-daily prolonged-release capsule of galantamine was developed [3].

Galantamine, as well as the other two ChEIs available currently (donepezil and rivastigmine), yields modest improvements in cognition, global performance and activities of daily living (ADL) compared with placebo treatment in subjects with varying degrees of AD severity [4]. However, a large heterogeneity in the response to, and long-term outcome of, ChEI treatment has been observed among individual patients. A meta-analysis demonstrated that larger ChEI doses are related to a better cognitive outcome [5] and an extension study suggested that effective dosages and sustained use might postpone the time to nursing home placement [6]. Furthermore, a more positive response to ChEI therapy has been reported among men compared with women [7,8].

Patients with AD are currently treated with ChEIs without actual knowledge of their concentration in plasma. Few studies have investigated whether drug concentration is a factor that influences the heterogeneity of the response to ChEI treatment. A relationship has been observed between the levels of AChE in the brain and in the cerebrospinal fluid (CSF) after treatment with galantamine. In addition, positive correlations have been found between AChE inhibition and the results of cognitive tests, mainly of those measuring attention [9]. Another AD study reported a dose-dependent increase in CSF AChE levels after ChEI therapy, and the increase was more prominent in patients showing a cognitive response according to the Mini-Mental State Examination (MMSE) test [10]. Those studies had the limitation of including small sample sizes and shorter follow-up intervals, and the fact that most patients originated from randomised trials.

It remains to be investigated whether a higher dose of galantamine or a higher drug plasma concentration correlates to a better cognitive and functional outcome in naturalistic patients with AD. Furthermore, other questions, such as whether drug plasma concentration differs between sexes or among patients with varying body mass index (BMI), need to be answered. Increased knowledge of these factors is clinically important and might lead to a better management of patients and enhanced drug efficacy. To address these questions, we investigated the plasma concentration levels of galantamine in a cohort of patients with AD in a routine clinical setting. The aims of this study were to investigate the associations between the plasma concentration of galantamine and sex, BMI, body weight, dose of galantamine and cognitive and functional responses to treatment.

## Methods

### Study and subjects

The patients were recruited prospectively from the Memory Clinic of the Skåne University Hospital in Malmö and enrolled in the Swedish Alzheimer Treatment Study (SATS). The study was undertaken to investigate the long-term effectiveness of ChEI treatment in naturalistic AD patients on various aspects of the disease (for example, cognitive, global and functional aspects). The SATS is a 3-year, open-label, non-randomised, multicentre study performed in a routine clinical setting. Most patients were in the mild-to-moderate stage of AD. The work-up at baseline included medical history, informant-based information, physical and neurological examination, extended cognitive testing, laboratory tests and computed tomography (CT) or magnetic resonance imaging (MRI) of the brain. Patients fulfilling the clinical criteria of dementia, as defined by the *Diagnostic and Statistical Manual of Mental Disorders*, *4^th ^edition *(DSM-IV) [11], and those of probable or possible AD, according to the National Institute of Neurological and Communicative Disorders and Stroke and the Alzheimer's Disease and Related Disorders Association (NINCDS-ADRDA) [12], were included in this study. Furthermore, the inclusion criteria were: AD patient older than 40 years, living at home at the time of diagnosis, having a responsible caregiver, and assessable with MMSE at baseline. Patients not fulfilling the diagnostic criteria for AD, those already undergoing active treatment with any ChEI drug, or individuals with contraindications for ChEI therapy were excluded from the study.

From January 2005 and for 2 years onwards, during their visits to the Memory Clinic the galantamine-treated SATS patients were consecutively asked to give blood samples for inclusion in this study. Eighty-four patients with AD provided repeat blood samples after treatment was initiated. The baseline characteristics of patients were recorded, including sex, apolipoprotein E (APOE) genotype, clinician's estimate of age at onset and duration of the disease, age at start of galantamine therapy, years of education and BMI.

### Outcome measures

The patients were assessed using cognitive, global and functional rating scales at the start of galantamine treatment, at 2 months (MMSE only) after the initiation of treatment and every 6 months over the course of 3 years. Trained dementia nurses obtained the ADL evaluation from an interview with the caregiver. In addition, BMI was calculated at every assessment using the formula body weight in kilograms/height in meters squared. The height of patients was measured once, whereas body weight in kilograms was measured at every assessment after the start of ChEI therapy. The reasons for dropping out of the study, such as adverse events, were recorded.

Cognitive ability was evaluated using the MMSE [13], with scores ranging from 0 to 30 (a higher score indicating less impaired cognition), and the Alzheimer's Disease Assessment Scale-cognitive subscale (ADAS-cog) [14], with a total score ranging from 0 to 70 (a higher score indicating more impaired cognition). Functional ability was measured using the Instrumental Activities of Daily Living (IADL) scale [15]. The latter consists of eight different items: telephone usage, shopping, food preparation, housekeeping, laundry, mode of transportation, responsibility for own medications and managing finances. Each item was scored from 1 (no impairment) to 3 to 5 (severe impairment), which resulted in a total score ranging from 8 to 31 points.

The rates of cognitive and functional change were calculated using three methods: 1) the change in score from the start of galantamine treatment (baseline) to the assessment with plasma extraction, divided by the number of months between these assessments; 2) the change in score from the previous assessment to the assessment with plasma extraction, divided by the number of months (usually 6) between these assessments; and 3) divided into two groups based on the patients' cognitive or functional rates of change per month during the study, that is, fast and slow decliners (cut-off median), using MMSE, ADAS-cog or IADL scores. To facilitate the comparison of MMSE, ADAS-cog and IADL scores, changes in score were converted to positive values, which were indicative of improvement, and negative values, which were indicative of decline.

### Galantamine treatment

After inclusion and baseline assessments, patients received galantamine treatment according to the approved product labelling, as in routine clinical practice. Patients were started on a dose of 8 mg per day, which was increased to 16 mg per day after 4 weeks of treatment, aiming at a further dose increase to 24 mg per day. In some cases, the dose was reduced because of side effects. All decisions regarding dosage were left to individual clinicians, as in routine clinical practice, and all dosage adjustments were recorded throughout the study. The patients paid for their medication in accordance with the standards of the Swedish healthcare system.

All patients and/or caregivers provided informed consent to participate in the study, which was conducted according to the provisions of the Helsinki Declaration and was approved by the Ethics Committee of Lund University, Sweden.

### Biochemical analysis

The plasma concentration of galantamine was determined using reversed-phase high-performance liquid chromatography with fluorescence detection [16]. The limit of detection of this method is 0.015 μmol/L and the coefficient of variation (CV) is 11.4% at a plasma level of 0.1 μmol/L and 4.3% at 2.0 μmol/L.

### Statistical analyses

The IBM SPSS statistics software version 19.0 (SPSS Inc., Chicago, IL, USA) was used to perform statistical analyses. The level of significance was defined as *P *< 0.05 if not otherwise specified. One-way analysis of variance (ANOVA) with the Bonferroni correction was used to compare the difference between the mean galantamine plasma concentrations according to galantamine dose. Independent samples *t*-tests were computed for the analysis of concentration or mean dose between sexes or APOE genotypes (two groups, presence or absence of ε4 allele). Pearson's correlation coefficient was calculated to investigate the presence of any linear associations between plasma concentration and the following variables: age at baseline, duration of AD, BMI, body weight, and cognitive and functional ability. Spearman's non-parametric correlation coefficient was used to analyse the ordinal variable drug dose.

Mixed-effects models with the galantamine plasma concentration as the dependent variable and study subject as a hierarchical variable (that is, to allow within-subject correlations) were used to study the multivariate impact on the abovementioned independent variables and the time from drug intake to plasma extraction as the fixed-effect terms. The random term in the models was an intercept with a variance components covariance matrix. Because of the strong linear correlation between the MMSE and ADAS-cog scores, these variables were entered into the models separately. Similarly, BMI and body weight were also entered individually. In a second mixed-effects model, the rates of change per month in MMSE (or ADAS-cog) and IADL scores were included, together with the previously mentioned independent predictors.

## Results

### Patient characteristics

The baseline characteristics and cognitive and functional outcomes for the entire cohort (*n *= 84) and for the 31 patients (37%) who were assessed after 3 years of galantamine treatment are described in Table 1.

**Table 1:** Patient characteristics

	Total cohort	3-year completers
Number of patients	84	31
Female sex, n (%)	60 (71%)	21 (68%)
APOE ε4 carrier, n (%)	57 (69%)	21 (68%)
		
Age at onset, years^a^	73.9 ± 7.1	73.3 ± 8.8
Age at start of treatment, years^a^	76.7 ± 7.0	76.9 ± 8.2
Illness duration, years^a^	3.0 ± 1.8	3.6 ± 2.3
Education, years^a^	10.1 ± 2.9	9.9 ± 2.9
		
MMSE score at baseline^a^	22.7 ± 4.0	23.2 ± 3.8
ADAS-cog score (0 to 70) at baseline^a^	17.1 ± 8.2	15.2 ± 8.3
IADL score at baseline^a^	13.4 ± 5.0	14.0 ± 6.0
		
MMSE score after 3 years^a ^(*n *= 31)		21.7 ± 4.6
ADAS-cog score after 3 years^a ^(*n *= 29)		20.7 ± 13.2
IADL score after 3 years^a ^(*n *= 24)		22.6 ± 5.8

^a^Mean ± standard deviation. ADAS-cog, Alzheimer's Disease Assessment Scale-cognitive subscale; APOE, apolipoprotein E; IADL, Instrumental Activities of Daily Living scale; MMSE, Mini-Mental State Examination.

### Galantamine plasma concentration

All patients had measurable levels of galantamine at all time points. The mean number ± SD (range) of blood samples per patient was 2.1 ± 1.3 (1 to 6). The number of samples obtained after each assessment was: 47 at 2 months, 41 at 6 months, 33 at 12 months, 18 at 18 months, 18 at 24 months, 12 at 30 months and 11 at 36 months of ChEI treatment; thus, 180 samples were acquired in total. The mean galantamine plasma concentration exhibited a strong positive linear association with drug dose (*r*_s _= 0.513, *P *< 0.001).

**Figure 1:**
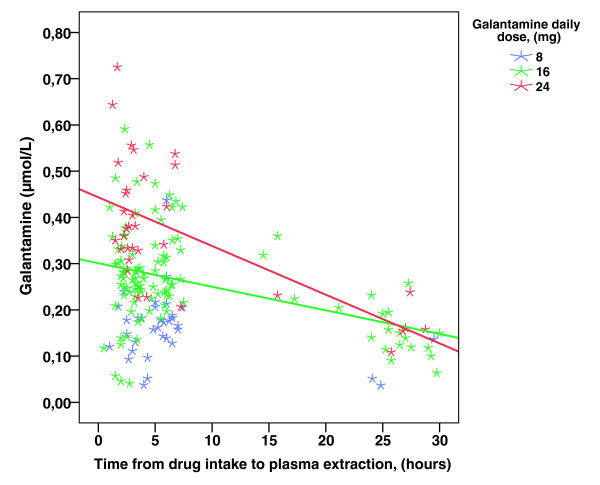
**Galantamine plasma concentration, dose and time from drug intake**. Time from drug intake to plasma extraction and galantamine plasma concentration level. The different galantamine doses are displayed: 8 mg, blue star; 16 mg, green star; 24 mg, red star. The lines illustrate a stronger negative linear association between galantamine plasma concentration and time from galantamine intake to plasma extraction among the individuals treated with 24 mg (*r *= -0.637, *P *< 0.001) and 16 mg (*r *= -0.414, *P *< 0.001) compared with those treated with 8 mg (*r *= -0.277, *P *= 0.113).

The mean ± SD dose of galantamine administered during the study was 14.0 ± 3.1 mg per day. Patients who received different daily doses of galantamine, namely 8 mg (*n *= 36), 16 mg (*n *= 108) and 24 mg (*n *= 34) (data were missing for two doses), differed significantly in mean ± SD plasma concentration (0.163 ± 0.073, 0.261 ± 0.105 and 0.368 ± 0.145 μmol/L, respectively; *P *< 0.001). Figure 1 suggests the presence of a stronger negative linear association between plasma concentration and time from drug intake to plasma extraction among the individuals treated with 24 mg (*r *= -0.637, *P *< 0.001) and 16 mg (*r *= -0.414, *P *< 0.001) compared with 8 mg of galantamine (*r *= -0.277, *P *= 0.113).

Age at baseline, duration of AD and cognitive and functional abilities at the start of treatment did not correlate with the plasma concentration or mean dose of galantamine. Four individuals withdrew from the study because of adverse events. The galantamine plasma concentrations of these individuals did not differ from those of the other patients.

#### Sex

Male patients had a significantly lower mean galantamine plasma concentration compared with female patients (0.218 ± 0.103 vs 0.277 ± 0.129 μmol/L, *P *= 0.005). No sex differences in the mean dose of galantamine were observed. No differences in the presence of the APOE ε4 allele, age at onset or at baseline, duration of AD, education in years and cognitive or functional abilities at baseline were detected between sexes.

#### BMI and body weight

**Figure 2:**
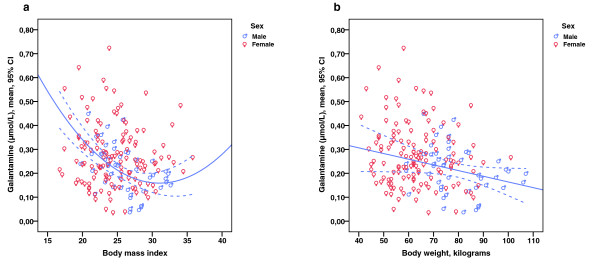
**Galantamine plasma concentration, sex, and body mass index or body weight**. **a) **Body mass index showed a quadratic relationship with the galantamine plasma concentration exclusively in male patients (R^2 ^= 0.239, *P *= 0.002). **b) **Body weight showed a negative correlation with the galantamine plasma concentration exclusively among male patients (*r *= -0.310, *P *= 0.034).

There were no linear, quadratic, or cubic relationships between the galantamine plasma concentration and BMI in the entire cohort. Investigation of the impact of sex revealed the presence of a negative linear association (*r *= -0.454, *P *= 0.001) between the galantamine plasma concentration and BMI exclusively in the male group. The addition of higher-degree polynomials slightly strengthened the explanation of the variance (linear R^2 ^= 0.206, *P *= 0.001; quadratic and cubic R^2 ^= 0.239, *P *= 0.002). The results of the quadratic model are illustrated (Figure 2a).

There was a negative linear association between the galantamine plasma concentration and body weight in the total cohort (*r *= -0.268, *P *< 0.001). As for the variable BMI, only the male group exhibited a significant linear relationship (*r *= -0.310, *P *= 0.034) (Figure 2b). The inclusion of quadratic and cubic terms marginally increased the degree to which the variance was explained. Therefore, the results of the linear model are presented.

There was no significant correlation between the change in the galantamine plasma concentration and the change in BMI or body weight in the entire cohort or when the sexes were analysed separately. However, the body weights of only 10 patients (all women) changed by more than ± 2 kilograms during the study. No linear association between the change in the plasma concentration and the change in BMI or body weight was found when only these individuals were analysed.

#### Cognitive and functional outcomes

The changes in MMSE, ADAS-cog or IADL scores from the start of ChEI therapy to the 2-month (MMSE only), 6-month or 12-month assessment, respectively, did not correlate with galantamine plasma concentration. No significant linear relationships were found between plasma concentration and cognitive or functional rate of change per month using any of the three calculation methods. Patients receiving different doses of galantamine (8, 16 or 24 mg daily) did not differ in cognitive or functional changes/month.

Although there were no significant associations between the rates of change and the plasma concentration, the mean differences (with the 95% CIs) in cognition and function from the start of galantamine treatment to the 2-month (MMSE only) and 6-month assessments are presented for each quartile of the plasma concentration (Table 2).

**Table 2:** Mean changes in cognitive and functional scores for each quartile of the galantamine plasma concentration

Galantamine plasma concentration after 2 months of treatment (*n *= 47)	Change in MMSE score after 2 months^a^	Galantamine plasma concentration after 6 months of treatment (*n *= 41)	Change in MMSE score after 6 months^a^	Change in ADAS-cog score after 6 months^a^	Change in IADL score after 6 months^a^
Quartile 1, < 0.143 μmol/L	0.42 (-1.17, 2.01)	Quartile 1, < 0.152 μmol/L	1.50 (-0.16, 3.16)	-1.90 (-5.51, 1.71)	-0.20 (-1.36, 0.96)
Quartile 2, 0.143 to 0.205 μmol/L	0.25 (-1.11, 1.61)	Quartile 2, 0.152 to 0.225 μmol/L	-0.36 (-1.33, 0.60)	0.36 (-1.12, 1.84)	-2.56 (-4.73, -0.38)
Quartile 3, 0.205 to 0.279 μmol/L	0.67 (-0.58, 1.92)	Quartile 3, 0.225 to 0.267 μmol/L	-0.60 (-2.19, 0.99)	-0.22 (-4.34, 3.90)	-2.89 (-6.36, 0.58)
Quartile 4, > 0.279 μmol/L	-0.36 (-1.81, 1.09)	Quartile 4, > 0.267 μmol/L	0.50 (-1.74, 2.74)	-0.20 (-2.25, 1.85)	0.00 (-1.15, 1.15)

^a^Mean (95% confidence interval). No significant differences in cognitive or functional changes were found among the patients in the different quartiles of the plasma concentration. ADAS-cog, Alzheimer's Disease Assessment Scale-cognitive subscale; IADL, Instrumental Activities of Daily Living scale; MMSE, Mini-Mental State Examination.

#### Multivariate analyses

Mixed-effects models using the galantamine plasma concentration as the dependent variable revealed that the drug dose (*P *< 0.001), time from drug intake (*P *< 0.001), and BMI (*P *= 0.021) or body weight (*P *= 0.002) were significant predictors of the drug concentration. The independent variables, sex, APOE genotype, age at baseline, duration of AD, and the MMSE (or ADAS-cog) and IADL scores at baseline were not significant predictors of the galantamine plasma concentration in the models. The cognitive and functional rates of change per month were also entered as independent predictors in the mixed-effects models, but these variables showed no significant relationship to the plasma concentration of galantamine.

## Discussion

In this study, we found that all patients included had a measurable concentration of galantamine at all assessment points. The mean galantamine plasma concentration exhibited a strong positive linear association with drug dose. Moreover, no sex differences regarding drug dose were observed. A negative linear association between the galantamine plasma concentration and BMI or body weight was found in the male, but not in the female group. The dose of galantamine, time from drug intake, and BMI or weight were predictive factors in the multivariate mixed-effects models in which the plasma concentration was used as the dependent variable. The galantamine plasma concentration showed no linear association with age, the cognitive or functional responses to ChEI treatment, or the longitudinal AD progression rate.

Currently, naturalistic patients with AD are treated with ChEIs without actual knowledge of their plasma or CSF concentration. Few studies have focused on whether drug concentration is a factor that affects the heterogeneity of the response to ChEI therapy. A small study of patients with mild AD reported that AChE levels in the CSF and in the brain are significantly correlated, both before and after treatment with galantamine [9]. A recent study reported positive and dose-related correlation between the plasma concentration of donepezil and increased AChE activity in the CSF. Treatment with galantamine also caused an increase in CSF AChE activity, but the increase was not dose dependent; however, the sample size was small (*n *= 15) and galantamine plasma concentration was not addressed in that study [17]. The increase in CSF AChE activity has been suggested as being greater in donepezil-treated compared with galantamine-treated patients [10,17] and sustained in rivastigmine-treated patients [18,19]. This was explained by different mechanisms of action of the substances used as reversible AChE inhibitors (donepezil, galantamine), and the substance used as a pseudo-irreversible AChE inhibitor (rivastigmine). Although the three ChEIs display various biochemical mechanisms and their effect on AChE activity might vary, the observed long-term treatment effect evaluated using cognitive and ADL scales does not seem to differ among the drugs [8,20,21]. Moreover, the plasma concentration of donepezil was 10 times higher in the blood than it was in the CSF; nevertheless, donepezil exhibited similar dose-dependent kinetics in that study [22]. In summary, these reports suggest that it is possible to identify a biochemical effect of ChEIs and to measure this effect in a dose-related manner.

In the present study, galantamine plasma concentration, but not AChE inhibition, was measured. Darreh-Shori *et al. *[22] suggested that the cognitive changes in patients with AD should be assessed in relation to AChE inhibition rather than ChEI dose. However, a meta-analysis showed that higher doses of donepezil and rivastigmine were associated with a better cognitive outcome. This dose effect was not described for galantamine [5]. However, a naturalistic long-term study performed by our group and including the three ChEIs reported that higher doses of ChEI were related to a more positive cognitive outcome, regardless of the type of drug [8]. The ChEIs might have different mechanisms of action and effects on AChE. Galantamine is a reversible AChE inhibitor with a competitive mode of action, which means that the degree of inhibition caused by the drug does not depend on the absolute concentration of galantamine; rather, it is more dependent on the association between the inhibitor and substrate concentration. Galantamine also potentiates cholinergic nicotinic neurotransmission by allosterically modulating the nicotinic receptors [2]. These mechanisms might cause the appearance of pharmacological effects early and at low concentrations; thus, a higher dose might not influence drug efficacy.

Men exhibited a lower galantamine plasma concentration compared with women in the present study, but no sex difference was found regarding drug dose. A negative linear correlation between the galantamine plasma concentration and BMI or body weight was found only in the male group, that is, men with higher BMI or weight exhibited lower galantamine plasma concentrations. This finding agrees with the results of a large study performed by Piotrovsky *et al. *[23], who observed that galantamine clearance was enhanced in men because of their greater body weight. In that study, and in ours when we used multivariate mixed-effects models, the dissimilarities in galantamine clearance were dependent on body weight but not on sex. However, a better response to ChEI treatment in men compared with women was reported previously [7,8].

A linear association between drug plasma concentration and short-term cognitive or functional response to galantamine treatment was not detected in the current study. In addition, the long-term AD progression rate did not correlate with galantamine plasma concentration. Previous studies that measured the levels of AChE activity in the CSF instead of the plasma concentration of ChEIs have reported contradicting results. Our findings are in line with those of Parnetti *et al. *[17], who showed no significant relationship between increased AChE activity and cognitive outcome. A linear association between AChE levels and change in MMSE score was found in one study including primarily donepezil-treated individuals [10]. Darreh-Shori *et al. *[9] reported that patients with high AChE inhibition showed a positive response, mainly in attention tests after galantamine therapy. Those 12 individuals with AD had less cognitive impairment and were younger and better educated than our SATS patients. Another study from that group including patients treated with donepezil reported a significant relationship between AChE inhibition in the CSF, and stabilised MMSE scores for up to 2 years [22]. These results indicate that the association between AChE activity or ChEI concentration, and cognitive outcome is not conclusive. The impact of AChE inhibition or plasma drug concentration on functional outcome has not been addressed previously. In this study, no relationships were observed between higher plasma concentrations of galantamine and better functional outcomes.

The strengths of the 3-year SATS programme are the prospective, well-structured, semi-annual follow-up assessments of a large AD cohort of ChEI-treated patients in clinical practice. A representative group of patients with mild-to-moderate AD and concomitant illnesses and medications from our Memory Clinic was included in this study. Conversely, some previous studies included a small sample size and the participants were enrolled from randomised clinical trials [9,10]. In our study, information from two cognitive tests (MMSE and ADAS-cog) and instrumental ADL ability, as well as body weight and BMI, were recorded at all evaluations. The short-term response to ChEI treatment and long-term rate of cognitive and functional changes were available measures. To the best of our knowledge, this is the first study investigating the effects of body weight, BMI, and sex on the galantamine plasma concentration in a routine clinical setting.

The patients in the SATS exhibit 100% compliance to ChEI treatment, and the levels of drug plasma concentration demonstrated a strong relationship to galantamine dose. The high adherence to treatment might depend on the regular 6-month visits to the Memory Clinic and the presence of an identified contact nurse for each patient. The SATS design represents high-quality individual care, continuity and security for the patients and their families and has currently evolved into a clinical follow-up programme that is applied to all patients with AD in our clinic.

Because the choice and dosage of ChEI for each individual patient was left entirely to the physician's decision, a common dose-optimising programme for this study was not applied. However, all dose adjustments were documented during the study. Plasma extraction was started while the SATS was ongoing. Therefore, the galantamine concentration was not measured for all patients at all time points, as it would be in a phase 1 trial. Our results reflect the outcomes for naturalistic AD patients in a memory clinic. Galantamine dose adjustment, changes in other medications and concomitant somatic disorders might have affected the drug plasma levels and cognitive and ADL abilities of patients over the course of the study. Another limitation of the current study is that AChE inhibition was not measured. However, a study of donepezil showed that AChE inhibition was dose dependent and strongly correlated with CSF and plasma drug concentration, with the exception that the concentration of donepezil in the CSF was approximately ten times lower than was the plasma level [22].

Future longitudinal studies including larger cohorts are warranted to investigate further the relationship between AChE inhibition, plasma or CSF concentration levels of the ChEI and cognitive and functional outcome. A dose-optimising schedule would be preferred to enhance drug efficacy. The mean dose of galantamine administered during the current study was 14 mg daily, indicating a potential for dose escalation.

## Conclusions

In conclusion, we found high compliance to treatment among all patients in this naturalistic AD study of galantamine. Drug plasma concentration and dose exhibited a strong linear relationship. Body weight or BMI, dose of galantamine, and time from drug intake were critical factors predicting the plasma concentration in the multivariate models. Obese men exhibited lower galantamine plasma concentration, indicating a potential for increasing the drug dose. Galantamine plasma concentration did not exhibit linear associations with age, cognitive and functional response to ChEI treatment or long-term progression rate; however, these outcomes might have been affected by the use of suboptimal doses of galantamine.

## Abbreviations

ACh: acetylcholine; AChE: acetylcholinesterase; AD: Alzheimer's disease; ADAS-cog: Alzheimer's Disease Assessment Scale-cognitive subscale; ADL: activities of daily living; ANOVA: analysis of variance; APOE: apolipoprotein E; BMI: body mass index; ChEI: cholinesterase inhibitors; CSF: cerebrospinal fluid; CT: computed tomography; CV: coefficient of variation; IADL: Instrumental Activities of Daily Living Scale; MMSE: Mini-Mental State Examination; MRI: magnetic resonance imaging; NINCDS-ADRDA: National Institute of Neurological and Communicative Disorders and Stroke and the Alzheimer's Disease and Related Disorders Association; SATS: Swedish Alzheimer Treatment Study.

## Competing interests

CW and AKW have no competing interests. EJ is employed as a medical advisor at Janssen-Cilag AB, Stockholm, Sweden. KB has served on scientific advisory boards for Innogenetics and Pfizer, as well as on a speakers' bureau for Janssen Alzheimer Immunotherapy.

## Authors' contributions

CW and AKW participated in the study, supervised the data collection, performed statistical analyses and interpreted the results. CW was responsible for the statistical design, for carrying out the statistical analyses and for drafting the paper together with EJ and AKW. EJ also initiated the protocol and case records forms and trained the staff in the logistic arrangement of the study. KB was responsible for the plasma analyses and critically revised the manuscript. All authors read and approved the final manuscript.
